# One Month Preexposure Prophylaxis Retention Rate and Associated Factors Among Adolescent Girls and Young Women Who Participated in the Namibia DREAMS Program (2018–2024)

**DOI:** 10.3390/idr17050110

**Published:** 2025-09-10

**Authors:** Enos Moyo, Endalkachew Melese, Hadrian Mangwana, Simon Takawira, Rosalia Indongo, Bernadette Harases, Perseverance Moyo, Ntombizodwa Makurira Nyoni, Kopano Robert, Tafadzwa Dzinamarira

**Affiliations:** 1 School of Nursing & Public Health, College of Health Sciences, University of Kwa-Zulu Natal, Durban 4041, South Africa; ntombizodwanyoni95@gmail.com; 2 Project HOPE—The People-to-People Health Foundation, Inc., Windhoek 9000, Namibia; emelese@projecthope.org; 3 Project HOPE Namibia, Windhoek 9000, Namibia; haddi81@gmail.com (H.M.); simontakawira11@gmail.com (S.T.); rindongo@projecthope.org (R.I.); bharases@projecthope.org (B.H.); 4 Medical Centre Oshakati, Oshakati 15001, Namibia; moyoperseverance@gmail.com; 5 School of Nursing, Faculty of Health, Humanities, & Social Sciences, Welwitschia University, Windhoek 9000, Namibia; rkpone2000@gmail.com; 6 School of Health Systems and Public Health, Faculty of Health Sciences, University of Pretoria, Pretoria 0028, South Africa; td2581@cumc.columbia.edu; 7 International Center for AIDS Care and Treatment Programs (ICAP) at Columbia University, Lusaka 10101, Zambia; 8 Africa Centre for Inclusive Health Management, Stellenbosch University, Stellenbosch 7600, South Africa

**Keywords:** early PrEP persistence, adolescent girls and young women, Namibia, HIV vulnerability, HIV prevention

## Abstract

**Background:** Daily oral preexposure prophylaxis (PrEP) is one strategy employed to decrease HIV transmission among adolescent girls and young women (AGYW). The Determined, Resilient, Empowered, AIDS-Free, Mentored, and Safe (DREAMS) program, funded by PEPFAR/USAID and implemented by the Project HOPE Namibia (PHN)-led consortium, provided services in the Khomas, Oshikoto, Zambezi, and Oshana regions. This study assessed the one-month PrEP retention rate among AGYW 15–24 and the associated factors. **Methods:** The program’s target populations for PrEP included AGYW aged 15–24 years who were at substantial risk for HIV, tested HIV-negative, and resided in the regions where the PHN-led consortium was implementing the DREAMS program. Data between 2018 and 2024 were exported from DHIS2 to IBM SPSS version 29 for secondary data analysis. We analyzed the data using Chi-squared tests and binomial and multinomial logistic regression. **Results:** Among the 17,277 participants newly initiated on oral PrEP and included in this study, only 2466 returned on time for their one-month appointment. The one-month PrEP retention rate among AGYW was 14.3%, 95% CI (13.8–14.8%). The most common reasons for PrEP discontinuation were traveling away from home, not needing PrEP anymore, forgetfulness, and side effects. Participants from Oshakati and Onandjokwe exhibited a higher likelihood of one-month PrEP retention. Additionally, participants who were in the programs for 7–12 months or over 36 months, who attended the safe space HIV prevention sessions, who were unaware of their partners’ HIV status, and who considered themselves at risk of HIV also exhibited a lower likelihood of one-month PrEP retention. In contrast, individuals who had 1–2 children and those who were either pregnant or breastfeeding exhibited a higher likelihood of one-month PrEP retention, (COR) = 1.28, 95% CI (1.15–1.43), and COR = 2.00, 95% CI (1.62–2.46), respectively. **Conclusions:** Targeted, innovative, and context-specific strategies should be developed to support AGYW in identifying their HIV risk and continuing the use of daily oral PrEP during periods of heightened risk. Additionally, prioritizing the introduction of discreet, long-acting PrEP options that require less frequent administration may better align with their needs and preferences.

## 1. Introduction

There has been a global decline in the number of new human immunodeficiency virus (HIV) infections. In 2023, approximately 1.3 million individuals were diagnosed with HIV globally [1]. The incidence of these infections is particularly notable among adolescent girls and young women (AGYW), especially in sub-Saharan Africa (SSA). In SSA, approximately 86% of new HIV infections occur in adolescents aged 15–19 years [2]. About 8% of the Namibian population is HIV-positive, and this translates to about 216,000 people living with HIV [3]. The Namibian annual HIV incidence among adults aged between 15 and 64 years is 0.36%. However, adolescents and young people aged 15–24 years old account for 35% of all new HIV infections, with AGYW aged 15–24 years of age disproportionately affected. The HIV incidence among AGYW is 0.9%, while that among adolescent boys and young men (ABYM) is 0.2% [3]. Multiple biological, socioeconomic, religious, and cultural factors contribute to the heightened risk among AGYW [4]. A comprehensive strategy is necessary to decrease HIV acquisition among AGYW. The strategy should encompass behavioral, biomedical, psychosocial, and structural interventions. Biomedical interventions include voluntary medical male circumcision (VMMC) for male partners, antiretroviral treatment as prevention (TasP), preexposure prophylaxis (PrEP), and post-exposure prophylaxis (PEP) [4].

PrEP entails the use of antiretroviral medications by HIV-negative individuals at high risk to prevent HIV infection. In 2016, the World Health Organization recommended oral preexposure prophylaxis (PrEP) for HIV prevention in all individuals at significant risk of HIV infection [5]. The recommended regimens for oral PrEP are a daily dose of tenofovir/emtricitabine or tenofovir/lamivudine as a single pill [5]. The number of individuals using oral PrEP worldwide rose from around 200,000 in 2017 to 3.5 million in 2023 [1]. A study conducted in South Africa indicated that the prevalence of oral HIV PrEP use for one month or less was 68.6% among AGYW. Persistence use for one to four months was 20.5% [6]. Another South African study indicated that the prevalence of PrEP continuation at one month was 63% [7]. One Namibian study revealed that 41.2% of AGYW were retained on oral PrEP for one month [8]. Multiple factors influence the retention of PrEP use among AGYW. Factors influencing the retention of PrEP use include having an HIV-positive partner [6], a high HIV-risk perception [6], a low HIV-risk perception [9], individualized service delivery [8], consistent interactions with healthcare providers [8], not having multiple partners [9], sharing experiences with peers [8], disclosing oral PrEP use [9], and establishing social networks and support systems for PrEP use [8].

AGYW in Namibia encounter multiple obstacles in obtaining HIV prevention services. A significant challenge is the requirement for adolescent girls to secure parental consent to access sexual and reproductive health services [10]. Some AGYW discontinue HIV PrEP use due to stigmatization from family and community members. Due to the presence of antiretroviral drugs in PrEP medications, AGYW indicated that family and community members assumed they were HIV-positive. AGYW using PrEP also encounter judgmental attitudes from healthcare professionals and community members regarding having sexual activity at a young age and before marriage [10]. Research on the impact of religion on PrEP retention among AGYW in Namibia is limited. However, a scoping review of studies in SSA indicates that religious opposition, stemming from the belief that PrEP promotion contradicts religious values, patriarchal norms, and conservative community beliefs, has exacerbated negative perceptions of PrEP and introduced further obstacles for AGYW [11].

PEPFAR and partners launched the Determined, Resilient, Empowered AIDS-free, Mentored, and Safe (DREAMS) program, which is a public-private partnership designed to reduce the rate of HIV among AGYW in 15 countries, including Namibia [9,12]. A consortium led by Project HOPE Namibia (PHN) successfully implemented the PEPFAR/USAID-funded DREAMS project from 4 June 2018 to 31 July 2023. PHN is currently leading a consortium to implement the follow-on activity, Reducing HIV Vulnerability: Integrated Child and Youth Health (Reach PHN), awarded on 31 July 2023. Unlike its predecessor, Reach PHN includes DREAMS and OVC (Orphan and Vulnerable Children) components. This manuscript presents the results of a secondary data analysis on the DREAMS program, implemented by the PHN-led consortia under the DREAMS project and Reach PHN, focusing on one month of PrEP retention among AGYW. This study aimed to assess the rate of one-month PrEP retention among AGYW and the associated factors. Upon identification of associated factors, strategies may be employed to encourage AGYW at high risk of HIV acquisition to continue the use of oral PrEP, potentially resulting in a decrease in new HIV infections within this target population in Namibia. This study addresses Sustainable Development Goals (SDGs) 3 and 5, which aim to ensure healthy lives, promote well-being for all ages, and reduce gender disparities and social injustices [13]. The study also responds to the World Health Organization’s recommendation of using a combination prevention approach to end the HIV epidemic by 2030 [14].

## 2. Methods

### 2.1. Study Design

We conducted a secondary analysis of programmatic data collected from AGYW who received clinical services through the Namibia DREAMS program, implemented by the PHN-led consortiums under both the DREAMS project and Reach PHN, between 2018 and 2024.

### 2.2. DREAMS Program Setting

The DREAMS project provided services in the Khomas, Oshikoto, and Zambezi regions, while the DREAMS component of Reach PHN expanded these services to the Oshana region. These regions exhibit significant socioeconomic differences. The Khomas region, where the capital city, Windhoek, is situated, has a population of 494,605. The majority of the people in the region are females (51.3%), stay in urban areas (97%), and have never been married (72.5%). About five percent of the population in the Khomas region has never had formal education. About 64% of the households in the Khomas region depend on wages and salaries as their primary source of income, and almost 5% live in poverty. Females make up 53.8% of the Oshana region’s population of 230,801. The majority of the population in the Oshana region stays in urban areas (53.2%) and has never been married (75.8%). About six percent of the population in the Oshana region has never had formal education. Almost 40% of the households in the Oshana region depend on wages and salaries as their primary source of income, and 21.1% live in poverty. The Oshikoto region has a population of 257,302, 50.5% of which are females. The majority of the population in the Oshikoto region has never been married (72.2%). Slightly less than a fifth (18.3%) of the population lives in urban areas, and about 12% have never had a formal education. A third (33.3%) of the households in the Oshikoto region depend on wages and salaries as their primary source of income, and almost 43% live in poverty. Slightly more than half (50.8%) of the Zambezi region’s population of 142,373 is made up of females. Over half (52.5%) of the Zambezi region’s population has never been married. About a third of the Zambezi region’s population lives in urban areas. Almost 38% of the households in the Zambezi region depend on wages and salaries as their primary source of income, and 39.3% live in poverty [15,16].

### 2.3. DREAMS Program Eligibility Criteria

The eligibility criteria for participation in the DREAMS program have been described elsewhere [17]. In summary, the criteria differed according to the age of the AGYW. Factors that were considered included a history of pregnancy; history of sexual, emotional, or physical violence; misuse of alcohol or other substances; being out of school; orphanhood; having multiple sexual partners; a history of sexually transmitted infection (STI) diagnosis or treatment; and having had transactional sex.

### 2.4. DREAMS Intervention

The DREAMS program, implemented by the PHN-led consortia under both the DREAMS project and Reach PHN, aims to prevent new HIV infections among AGYW. The DREAMS program addresses factors that increase HIV vulnerability among AGYW, such as gender-based violence, economic exclusion, and limited access to health services. This is achieved by delivering a core package of age-appropriate “primary” interventions for all AGYW, alongside “secondary” interventions tailored to DREAMS-eligible AGYW aged 10 to 24 years. Additionally, interventions aimed at strengthening families and reducing risks among sexual partners of AGYW are provided. Standardized PEPFAR eligibility criteria are used to determine eligibility for the DREAMS program, with interventions guided by the PEPFAR Namibia DREAMS layering table.

Upon enrollment into DREAMS, AGYW were grouped based on age (10–12, 13–14, 15–19, and 20–24) and location, referred to as “safe spaces”. Safe spaces are judgment-free, secure, and private locations where AGYW groups meet regularly under trained mentors’ guidance. These spaces included school classrooms, rooms or open areas within local non-governmental organizations or community centers, rooms in technical colleges or universities, adolescent service delivery rooms in health centers, and church meeting rooms. These locations functioned as safe spaces only during designated DREAMS meetings or service delivery days and times and were not open for walk-ins. Safe spaces were selected through a mapping exercise that included interviews with AGYW and community members to determine where AGYW felt most comfortable gathering, how far they were willing to travel, and what resources were already available at potential sites. Within these safe spaces and other DREAMS service delivery points, including health facilities, AGYW received the core primary and secondary package of interventions.

For AGYW aged 10–12 and 13–24 years, a minimum of 16 and 19 sessions of HIV and violence prevention education, respectively, were delivered using the Window of Hope (WoH) curriculum. They also received social asset-building (WoH), HIV risk assessments, and screening for Gender-Based Violence (GBV) and Sexual and Reproductive Health (SRH) needs [18]. AGYW aged 15–19 received a minimum of 10 sessions of HIV and violence prevention education using the My Future My Choice (MFMC) curriculum, along with social asset-building, risk assessments for HIV, and screening for GBV and SRH needs. Education on condoms, PrEP, and contraceptive methods was also provided [18]. AGYW aged 20–24 received similar interventions as those provided to the 15–19 age group.

Assessments were conducted every six months to identify secondary intervention needs. These secondary interventions vary by age group but generally include PrEP, post-violence care, contraceptive methods, economic strengthening interventions, and educational support.

### 2.5. DREAMS Approach to PrEP Provision

DREAMS delivered nurse-led, comprehensive PrEP services through a hybrid model, combining health facility-based delivery via adolescent and youth-friendly corners and community-based service delivery (fixed/mobile) through a facility-linked outreach approach. Existing structures such as schools, churches, community centers, and other preferred locations, along with tents and vehicles stationed outside fixed community sites, were utilized to ensure private and confidential community-based PrEP service delivery. Through interpersonal communication, AGYW are informed in advance about the schedule for community-based PrEP services. The hybrid model allows AGYW to transition between service delivery models at any time during the initiation or follow-up phase. The DREAMS curriculum, flipcharts, pamphlets, posters, social media posts, and radio spots were utilized to generate demand. This information was shared by DREAMS ambassadors, peer educators, community mobilizers, community care workers (CCWs), and nurses.

DREAMS nurses trained in PrEP screen all sexually active girls aged 15 to 24 for PrEP eligibility using the National Risk Assessment and Clinical Eligibility Screening (RACES) tool at all service delivery points. Per national guidelines, AGYW were considered at substantial risk for HIV if they fulfilled one or more of the following criteria: sero-different relationship with a partner who is not confirmed as virologically suppressed (i.e., partner with VL > 40 copies/mL), sero-different relationship (regardless of VL of the partner) and desire to conceive, pregnant or breastfeeding women (PBFW), partner(s) of unknown HIV status, engaged in transactional or intergenerational sex, recent STI (within the last 3 months) and/or recurrent STIs, multiple and/or concurrent sexual partners, history of inconsistent or no condom use, recurrent PEP user, history of having sex whilst under the influence of alcohol or recreational drugs, abusive relationships (GBV/IPV), and strongly feel at substantial risk of HIV infection.

AGYW who were at substantial risk for HIV and willing to use PrEP were provided with HIV testing services (HTS). Following a negative HIV rapid test, nurses collected and sent specimens for additional laboratory tests, including creatinine, hepatitis B surface antigen (HBsAg), RPR, and a pregnancy test at baseline. PrEP was then initiated on the same day. AGYW on PrEP were contacted if test results required further action, confirmation, or treatment. Follow-up visits and laboratory tests were conducted per national guidelines. Client data for initiation and follow-up services were documented using the Ministry of Health and Social Services (MHSS) PrEP client record and the clinical register developed by the PHN-led consortium. Medical supplies were sourced from hub health facilities using the existing supply chain management system. The strategies implemented to improve the continued use of PrEP are summarized in Table 1.

### 2.6. Data Collection

PHN and its partners employed a standardized data collection instrument to determine participants’ HIV status and risk to maintain consistency and comparability across sites. Site-level personnel utilized the District Health Information Software 2 (DHIS2) (version 40, HISP Centre, Oslo, Norway) Android Application for regular data collection from source documents, including PrEP client records, clinical registers, and PrEP tracing forms, alongside other integrated applications for beneficiary feedback, site supervision documentation, and sentinel survey data collection. The PrEP tracing form documents the outcomes of tracing AGYW who discontinued PrEP, with tracing conducted in person or via phone call. Site-level personnel recorded all data in DHIS2, assisted by data entry clerks (DECs) during point-of-service delivery. After data entry into DHIS2, district and regional teams performed routine quality control checks to ensure completeness and accuracy. We abstracted data on the sociodemographic characteristics and HIV risk factors of participants from the DREAMS participant records and the PrEP clients’ records. Individual-level data were linked across sources using the unique DREAMS participant identification number.

### 2.7. Outcome Variable

The dependent variable for this study was PrEP retention at one month. Participants who went back to the healthcare facilities to collect their PrEP drugs within 30 days after initiation were recorded as having been retained, while those who did not come back were recorded as having not been retained. This definition is consistent with previous studies [19,20].

### 2.8. Explanatory Variables

Seventeen explanatory variables were used in this study. The explanatory variables were categorized into nine participant characteristics and eight HIV risk factors. Participant characteristics were the age group, the number of biological children the AGYW had, the marital status, the district of residence, the year of enrollment into the DREAMS program, and the number of months in the DREAMS program. Additional participant characteristics were family planning utilization, whether the participant was pregnant or breastfeeding, and whether she had participated in the safe space interventions. The HIV risk factors were the partner’s HIV status, inconsistent or no condom use, recent or recurrent STIs, recurrent PEP use, and being a victim of sexual violence. Furthermore, the other HIV risk factors were having had sex under the influence of alcohol or drugs, having multiple or recurrent partners, and considering oneself at risk of HIV.

### 2.9. Data Quality Assurance

Our digital system enabled the automatic generation of BioID (Unique Identifier Code), implemented automated skip rules, and performed validation checks for variables, including age and sex, and constraints for mandatory questions. The digital system reduced transcription errors, enhancing data completeness and quality. Data quality assurance (DQA) mechanisms comprised periodic programmatic spot checks, desk reviews, data quality reviews, and field monitoring activities conducted by district and regional teams to verify that reported data adhered to minimum quality standards and incorporated digital enhancements.

### 2.10. Criteria for Inclusion in Data Analysis

Among the 31,276 AGYW who received clinical services through the DREAMS program from 2018 to 2024, we excluded those aged between 10 and 14 from the analysis since PrEP is contraindicated in this age group. After removing the 10–14-year-old AGYW, we remained with 30,230. After excluding AGYW who were continuing, restarting, or had discontinued PrEP, we remained with 24,190 who were newly initiated. Among those newly initiated, we excluded participants not due for a one-month follow-up visit, leaving 20,500 remaining. After excluding participants with missing information, we conducted data analysis on the remaining 17,227 participants.

### 2.11. Data Analysis

Data were exported from DHIS2 to IBM SPSS version 29 (Armonk, New York, NY, USA) for analysis. This study defined one-month retention for PrEP as AGYW who return within 14 days of the scheduled one-month visit. Descriptive statistics encompassing percentages and frequencies were employed to analyze nominal and ordinal data. Chi-square tests evaluated the associations between early PrEP persistence and participant characteristics, as well as their HIV risk factors. Characteristics identified as statistically significant through Chi-square tests were further analyzed using bivariate logistic regression to evaluate the strength of their associations with early PrEP persistence. Characteristics showing statistically significant associations with early PrEP persistence, indicated by a *p*-value below 0.05 in bivariate logistic regression, were subsequently employed in multivariate logistic regression to determine the adjusted odds ratios.

### 2.12. Ethical Considerations

The DREAMS program, implemented by the PHN-led consortia under both the DREAMS project and Reach PHN, received approval from the Namibian MHSS, the Ministry of Education, Arts, and Culture (MoEAC); the Ministry of Gender Equality, Poverty Eradication, and Social Welfare (MGEPESW); and the Ministry of Sport, Youth, and National Service (MSYNS). PHN enforces a robust privacy management framework by requiring all personnel to sign a non-disclosure agreement, thus ensuring the protection of all collected data. Access to DHIS2 was allocated according to specific roles and requirements. Each user received a distinct username along with password-protected login credentials. When data sharing was required, de-identified or aggregated data were utilized. All minors in the program provided assent, and their parents or caregivers granted consent. AGYW of legal age completed a consent form. Since we used anonymous programmatic data, we did not apply for an institutional body clearance for the secondary data analysis.

## 3. Results

### 3.1. Characteristics of Participants

Among the 17,277 AGYW who were included in the analysis, the majority were in the age group 20–24 years (*n* = 9925; 57.6%), had no children (*n* = 14,375; 83.4%), had not participated in the safe space interventions (*n* = 14,773; 85.8%), were not using any family planning method (*n* = 12,319; 71.5%), were neither pregnant nor breastfeeding (*n* = 16,695; 96.9%), were less than six months in the program (*n* = 11,674; 67.8%), and did not return for follow-up one month after initiation (*n* = 14,761; 85.7%). More details are in Table 2.

### 3.2. HIV Risk Factors Among Participants

Most participants included in this secondary analysis considered themselves at risk of HIV (*n* = 12,221; 70.9%), did not use condoms or inconsistently used them (*n* = 11,075; 64.3%), and did not know their partner’s HIV status (*n* = 9271; 53.8%). A few of the participants had multiple or recurrent sexual partners (*n* = 273; 1.6%), had recent or recurrent STIs (*n* = 127; 0.7%), had sex under the influence of alcohol or drugs (*n* = 96; 0.6%), were victims of sexual violence (*n* = 33; 0.2%), and were recurrent post-exposure prophylaxis users (*n* = 19; 0.1%). More details are in Table 3.

### 3.3. One-Month PrEP Retention Rate

Among the 17,277 AGYW who were newly initiated on oral PrEP and included in the analysis, 14,761 (85.7%) did not return on time for a one-month scheduled appointment, 95% confidence interval (CI) (85.2–86.2%), while 2466 (14.3%) returned, 95% CI (13.8–14.8%).

### 3.4. Reasons for PrEP Discontinuation

Among the 1506 participants who provided reasons for discontinuing PrEP, almost half reported traveling away from home and not needing PrEP anymore because of knowing a partner’s HIV status or not having a sexual relationship. Other reasons were forgetfulness, side effects, a lack of transport money, and a long distance to a health facility. More details are in Table 4.

### 3.5. Determinants of One-Month PrEP Retention Among Participants

Chi-square tests revealed statistically significant associations between one-month PrEP retention and the district, the year of enrollment, the number of months in the program, safe space participation status, knowledge of a partner’s HIV status, consistency of condom usage, marital status, the number of biological children, pregnancy or breastfeeding status, and consideration of self-risk of HIV (*p* < 0.05). However, there was no association between one-month PrEP retention and age group, the use of a family planning method, recent or recurrent STIs, recurrent PEP use, having had sex under the influence of alcohol or drugs, or having multiple or concurrent partners. Participants with 1–2 children had a higher likelihood of one-month PrEP retention than those without children, crude odds ratio (COR) = 1.28, 95% CI (1.15–1.43). However, this association did not hold in multivariate analysis. Participants from Oshakati and Onandjokwe were more likely to be retained on PrEP for one month than those in Katima Mulilo, with COR = 3.24, 95% CI (2.74–3.84), and COR = 1.22, 95% CI (1.05–1.41), respectively. Participants who enrolled in 2019, 2020, 2021, 2022, and 2023 had a lower likelihood of one-month PrEP retention than those who enrolled in 2024, adjusted odds ratio (AOR) = 0.81, 95% CI (0.67–0.99), AOR = 0.40, 95% CI (0.34–0.47), AOR = 0.16, 95% CI (0.14–0.20), AOR = 0.34, 95% CI (0.29–0.40), and AOR = 0.11, 95% CI (0.08–0.15), respectively. Participants 7–12 months and >36 months in the programs had a lower likelihood of one-month PrEP retention than those under six months, AOR = 0.59, 95% CI (0.47–0.74), AOR = 0.26, 95% CI (0.21–0.33), respectively. Participants who did not know their partners’ HIV status and those who had participated in safe space HIV prevention sessions had a lower likelihood of one-month PrEP retention than their counterparts, AOR = 0.90, 95% CI (0.81–0.99), and AOR = 0.66, 95% CI (0.56–0.78), respectively. Participants who did not or inconsistently used condoms and who considered themselves at risk of HIV were less likely to be retained on PrEP for one month, COR = 0.71, 95% CI (0.65–0.77), and AOR = 0.86, 95% CI (0.78–0.96), respectively. Pregnant or breastfeeding participants were more likely to be retained on PrEP for one month than those with neither, COR = 2.00, 95% CI (1.62–2.46), and COR = 2.51, 95% CI (1.24–5.06), respectively. More details are in Table 5.

## 4. Discussion

The study found that the one-month PrEP retention rate among AGYW who participated in the DREAMS program implemented by PHN was 14.3%. The most common reasons for PrEP discontinuation were traveling away from home, not needing PrEP anymore, forgetfulness, side effects, a lack of transportation money, and a long distance to a health facility. Participants from Oshakati and Onandjokwe, those who enrolled in the programs between 2019 and 2023, who were in the programs for 7–12 months or over 36 months, who attended the safe space HIV prevention sessions, who were unaware of their partners’ HIV status, and who considered themselves at risk of HIV, exhibited a lower likelihood of one-month PrEP retention. Conversely, individuals who had 1–2 children and those who were either pregnant or breastfeeding exhibited a higher likelihood of one-month PrEP retention.

In this study, one-month PrEP retention was lower than the 41.2% rate reported in a study conducted in Namibia [8] and marginally higher than the 12.4% reported in another Namibian study [21]. The rate observed in the current study was lower than that reported in a South African study, which indicated a prevalence of 20.5% [6]. The retention rate observed in this study may have been lower than that reported in the previous Namibian study, as our research encompassed participants from six regions of the country. The earlier study was limited to participants in Windhoek [8]. The DREAMS programs implemented by PHN lacked nurses at all healthcare facilities in the districts, potentially resulting in the non-capture of AGYW who chose to have their PrEP follow-up at facilities without a PHN-employed nurse. In addition to non-capture, alterations in risk status may have affected the proportion of AGYW who persisted with PrEP after one month. A study in Kenya indicated that the primary reason for PrEP discontinuation was the participants’ assessment of no longer being at risk [22]. Innovative strategies are necessary to assist AGYW in recognizing their HIV risk, thereby enabling informed decisions regarding PrEP continuation [23]. In this study, 13.5% of the participants discontinued PrEP due to side effects. The rate in this study is half of the 27% reported in a study conducted in South Africa [24]. It is crucial to counsel PrEP users on the expected side effects, since some of the side effects are transient [25].

Our findings indicate that early PrEP persistence was higher in Oshakati and Onandjokwe than in Katima Mulilo. These variations may be attributed to programmatic differences among the regions. Internal monitoring and evaluation of the program revealed that peer educators and DREAMS ambassadors, responsible for educating on, advocating for, and championing PrEP as well as forming and running PrEP clubs in Oshakati and Onandjokwe, were more effective in conducting their duties and offered support and encouragement to AGYW to persist in PrEP use. The PrEP clubs contributed to a reduction in community stigma since AGYW in the clubs disclosed that they were using PrEP and shared information on PrEP and HIV prevention. Previous research indicates that youth PrEP clubs foster supportive environments that promote PrEP adherence and diminish community stigma [26,27,28,29]. Studies conducted in South Africa revealed that AGYW preferred attending youth PrEP clubs because they received social support from their peers and discussed other life problems that affect them [26,27]. We, therefore, recommend that Namibia provide incentives to peer Educators and DREAMS ambassadors so they can be more efficient in their duties and increase PrEP retention among AGYW. Health education on PrEP should also be offered to communities through mass and social media, as well as by healthcare professionals and community health workers, to reduce stigma against AGYW who use PrEP.

Participants who enrolled between 2019 and 2023 and those who engaged in the programs for over 36 months exhibited a lower likelihood of one-month PrEP retention. The findings may be attributed to the possibility that participants who engaged with the program for an extended duration better understood their risks, leading them to stop PrEP usage beyond one month. Moreover, the DREAMS component of Reach PHN, which began delivering PrEP services in 2024, has refined its PrEP retention strategy based on the lessons learned from the DREAMS project. However, it is essential to note that the duration of program participation is measured from the day an AGYW is enrolled in the DREAMS program without accounting for their actual participation status (active vs. inactive). As a result, many AGYW with over 36 months of program participation may not have been active participants for the entire duration. It is essential to recognize that PrEP retention may not accurately indicate PrEP adherence. A study in Kenya indicated that many AGYW continued in the PrEP program but did not adhere to the regimen sufficiently to gain benefits [30]. Short message service (SMS) support and refill reminders, which have been used with success in South Africa [31], should also be implemented in Namibia to enhance PrEP adherence and retention among AGYW, potentially reducing new HIV infections. Persistent non-adherent AGYW may benefit from long-acting PrEP injectables upon their availability because they are discrete, do not require daily intake, and address pill fatigue [32]. We, therefore, advocate for the Namibian government to include long-acting PrEP injectables in the country’s HIV prevention guidelines, especially for high-risk populations like AGYW.

Participants who were unaware of their partners’ HIV status and those who considered themselves at risk of HIV exhibited a lower likelihood of one-month PrEP retention. The findings of the current study are at variance with those of a study conducted in Kenya, which indicated that AGYW who viewed themselves as at high risk for HIV infection continued their participation in the PrEP program [30]. We expected a higher likelihood of one-month PrEP retention among participants at high risk of HIV because they may be motivated to continue taking PrEP beyond one month to prevent HIV infection. Addressing HIV stigma in the Namibian communities through education and advocacy campaigns may help improve HIV status disclosure among sexual partners.

Participants who attended the safe space HIV prevention sessions had a lower likelihood of one-month PrEP retention. This may be attributed to the recruitment criteria for the intervention. Some of the participants were misusing alcohol and substances or had experienced physical, emotional, or sexual violence. As noted in previous studies, these people are unlikely to be retained in PrEP care [33,34]. It is vital for the Namibian government to provide more alcohol and substance misuse rehabilitation facilities for AGYW in all regions of the country, since AGYW who recover from alcohol and substance misuse have a higher chance of being retained in PrEP care. Additionally, healthcare facilities should be adequately staffed with social workers and psychologists to cater to AGYW who have experienced physical, emotional, and sexual trauma.

This study revealed that participants who were either pregnant or breastfeeding and those who had 1–2 children were more likely to be retained on PrEP for one month. Pregnant or breastfeeding AGYW might have been motivated to continue on oral PrEP beyond one month to safeguard their health and ensure the birth of HIV-negative children [35]. The advantages of PrEP during pregnancy and breastfeeding must be highlighted during antenatal and postnatal care in Namibia to enable AGYW to make informed choices. AGYW with 1–2 children might also have been motivated to continue taking PrEP beyond one month to remain healthy and care for their children.

A notable concern was the low utilization of family planning methods among AGYW initiated on PrEP, likely due to frequent stockouts of commodities and a lack of awareness that PrEP does not prevent pregnancy or other STIs. These findings highlight the urgent need for improved sexual and reproductive health education and interventions to ensure commodity security.

The strength of this study is its use of a large sample size from six regions in Namibia, which facilitates the generalization of the findings for the country. The digital data collection reduced the probability of errors. Nevertheless, except for the dependent variable, one-month PrEP retention, all other responses were self-reported, which may have been subject to social desirability bias. An additional limitation was that only variables included in the dataset were used in the study, resulting in other important variables influencing PrEP retention being excluded.

## 5. Conclusions

The study indicated that merely 14.3% of AGYW initiated on PrEP through the DREAMS program implemented by PHN returned for one-month scheduled follow-up visits. The study revealed that factors such as participants’ year of enrollment, duration in the program, involvement in safe space HIV prevention sessions, awareness of partners’ HIV status, pregnancy or breastfeeding status, and the number of biological children affected one-month PrEP retention. Targeted, innovative, and context-specific strategies should be developed to support AGYW in identifying their HIV risk and continuing the use of daily oral PrEP during periods of heightened risk. Additionally, prioritizing the introduction of discreet, long-acting PrEP options that require less frequent administration may better align with their needs and preferences. The use of family planning methods was notably low among AGYW initiated on PrEP, highlighting the need for improved SRH education and urgent interventions to ensure commodity security. A future qualitative study is needed to explain AGYW’s reasons for discontinuing PrEP.

## Figures and Tables

**Table 1:** Strategies implemented to improve the continued use of PrEP.

Interventions at ‘’Typical’’ Sexual Partners’ Level	
Increasing knowledge about PrEP	▪ Male engagement session knowledge content
	▪ Pocket-size flyer
	▪ Webpage and social media
Promote PrEP as a relationship positive	▪ Social media
	▪ Poster
	▪ Role plays as part of the male engagement session
	▪ Pocket-size flyer
Interventions at AGYW/Peer Level	
Promote alignment of PrEP with life goals	▪ Flipchart
Improve relationship communication about PrEP	▪ Flipchart
Increase PrEP continuation	▪ Repackage of PrEP: use of pencil bags and pill bottles ▪ PrEP Club ▪ Provision of dignity packs (soap, menstrual pads, condoms, toothbrush, toothpaste, deodorant, stickers, and sweets) as an incentive if AGYW returns for follow-up ▪ PrEP Supporters ▪ One-week follow-up call ▪ Support for the introduction of long-acting injectable cabotegravir (CAB-LA) ▪ Support for implementing the revised PrEP standard operating procedures (SOP)
Interventions at the Parents/Community Level	
Positive messaging about PrEP	▪ Radio spot
	▪ Social media
	▪ Webpage
Engaging parents to create an enabling environment for AGYWs’ PrEP use	▪ Supplementary content on PrEP for families and parents

**Table 2:** Frequency distribution of characteristics of participants.

Characteristics	Frequency, *n* (%)
Age group (years)	
15–19	7302 (42.4)
20–24	9925 (57.6)
Number of biological children	
0	14,375 (83.4)
1–2	2721 (15.8)
≥3	131 (0.8)
Marital status	
Single	5330 (30.9)
Married	35 (0.2)
Missing	11,862 (68.9)
Participated in safe space interventions	
Yes	2454 (14.2)
No	14,773 (85.8)
District	
Katima	3057 (17.7)
Omuthiya	1868 (10.8)
Onandjokwe	2703 (15.7)
Oshakati	989 (5.8)
Tsumeb	1144 (6.7)
Windhoek	7466 (43.3)
Year of enrollment	
2018	263 (1.5)
2019	1654 (9.6)
2020	4761 (27.6)
2021	4039 (23.4)
2022	2842 (16.5)
2023	1222 (7.2)
2024	2446 (14.2)
Months in the program	
0–6 months	11,674 (67.8)
7–12 months	1209 (7.0)
13–24 months	1760 (10.2)
25–36 months	926 (5.4)
>36 months	1658 (9.6)
Using family planning method	
Yes	4908 (28.5)
No	12,319 (71.5)
Pregnant or breastfeeding	
Pregnant	494 (2.9)
Breastfeeding	38 (0.2)
Neither	16,695 (96.9)
Followed-up at 30 days of initiation	
Yes	2466 (14.3)
No	14,761 (85.7)

**Table 3:** Frequency distribution of HIV risk factors among participants.

Characteristics	Frequency, *n* (%)
Unknown partner’s HIV status	
Yes	9271 (53.8)
No	7956 (46.2)
Inconsistent or no condom use	
Yes	11,075 (64.3)
No	6152 (35.7)
Recent or recurrent STIs	
Yes	127 (0.7)
No	17,100 (99.3)
Recurrent post-exposure prophylaxis (PEP) use	
Yes	19 (0.1)
No	17,208 (99.9)
Having had sex under the influence of alcohol or drugs	
Yes	96 (0.6)
No	17,131 (99.4)
A victim of sexual violence	
Yes	33 (0.2)
No	17,194 (99.8)
Has multiple or recurrent sexual partners	
Yes	273 (1.6)
No	16,954 (98.4)
Considers oneself at risk of HIV	
Yes	12,221 (70.9)
No	5006 (29.1)

**Table 4:** Frequency distribution of reasons for PrEP discontinuation among participants.

Reason for Discontinuation of PrEP	Frequency *n* (%)
Traveling away from home	403 (26.8)
Did not need PrEP anymore	371 (24.7)
Forgetfulness	228 (15.1)
Side effects	206 (13.5)
A lack of transport money	105 (7.0)
Long distance to the health facility	63 (4.3)
Inconvenient appointment dates/days	34 (2.2)
An employer refusing to permit an employee to go to the clinic	21 (1.4)
Did not understand the instructions on PrEP	20 (1.3)
Pill burden	19 (1.3)
Tired of taking PrEP	13 (0.9)
A lack of food	12 (0.8)
No family support	11 (0.7)
Total	1506 (100%)

**Table 5:** Determinants of early PrEP persistence among participants.

Characteristics	Crude Odds Ratios	95% CI *	Adjusted ** Odds Ratios	95% CI *	Chi-Square Test *p*-Value
Age group (years)					0.21
15–19	NC	NC	NI	NI	
20–24	NC	NC	NI	NI	
Marital status					**<0.01**
Single	0.72	0.33–1.60	NI	NI	
Married	Reference	Reference	Reference	Reference	
Number of biological children					**<0.01**
0	Reference	Reference	Reference	Reference	
1–2	**1.28**	**1.15–1.43**	0.94	0.83–1.07	
≥3	0.99	0.60–1.64	0.70	0.41–1.18	
District					**<0.01**
Katima Mulilo	Reference	Reference	Reference	Reference	
Omuthiya	1.17	0.99 -1.38	1.03	0.87–1.24	
Onandjokwe	**1.22**	**1.05–1.41**	0.88	0.71–1.08	
Oshakati	**3.24**	**2.74–3.84**	1.03	0.88–1.22	
Tsumeb	1.01	0.82–1.23	1.14	0.93–1.39	
Windhoek	0.92	0.81–1.04	0.97	0.85–1.11	
Year of enrollment					**<0.01**
2018	0.79	0.60–1.05	1.44	1.04–2.00	
2019	**0.49**	**0.42–0.56**	**0.81**	**0.67–0.99**	
2020	**0.29**	**0.26–0.33**	**0.40**	**0.34–0.47**	
2021	**0.13**	**0.11–0.15**	**0.16**	**0.14 -0.20**	
2022	**0.30**	**0.26–0.35**	**0.34**	**0.29–0.40**	
2023	**0.10**	**0.08–0.14**	**0.11**	**0.08–0.15**	
2024	Reference	Reference	Reference	Reference	
Months in the programs					**<0.01**
0–6 months	Reference	Reference	Reference	Reference	
7–12 months	**0.45**	**0.37–0.56**	**0.59**	**0.47–0.74**	
13–24 months	**0.74**	**0.63–0.86**	1.11	0.94–1.30	
25–36 months	0.86	0.71–1.04	0.92	0.75–1.27	
>36 months	**0.36**	**0.30–0.44**	**0.26**	**0.21–0.33**	
Participated in safe space HIV prevention sessions					**<0.01**
Yes	**0.49**	**0.42–0.57**	**0.66**	**0.56–0.78**	
No	Reference	Reference	Reference	Reference	
Using a family planning method					0.83
Yes	NC	NC	NI	NI	
No	NC	NC	NI	NI	
Partner’s HIV status is unknown					**<0.01**
Yes	**0.67**	**0.61–0.73**	**0.90**	**0.81–0.99**	
No	Reference	Reference	Reference	Reference	
Inconsistent or no condom use					**<0.01**
Yes	**0.71**	**0.65–0.77**	1.04	0.93–1.16	
No	Reference	Reference	Reference	Reference	
Recent or recurrent STIs					0.96
Yes	NC	NC	NI	NI	
No	NC	NC	NI	NI	
Recurrent post-exposure prophylaxis (PEP) use					0.64
Yes	NC	NC	NI	NI	
No	NC	NC	NI	NI	
Having had sex under the influence of alcohol or drugs					0.17
Yes	NC	NC	NI	NI	
No	NC	NC	NI	NI	
Has multiple or concurrent sexual partners					0.22
Yes	NC	NC	NI	NI	
No	NC	NC	NI	NI	
Considers oneself at risk of HIV					**<0.01**
Yes	**0.66**	**0.60–0.72**	**0.86**	**0.78–0.96**	
No	Reference	Reference	Reference	Reference	
Pregnant or breastfeeding					**<0.01**
Pregnant	**2.00**	**1.62–2.46**	1.00	0.79–1.27	
Breastfeeding	**2.51**	**1.24–5.06**	1.34	0.63–2.86	
Neither pregnant nor breastfeeding	Reference	Reference	Reference	Reference	

NC—not computed; NI—not included; * CI is the 95% confidence interval; ** adjusted for district, year of enrollment, number of months in the programs, participation in the safe space HIV prevention sessions, consistency of condom use, number of biological children, knowledge of partner’s HIV status, consideration of HIV self-risk, and pregnancy or breastfeeding status; Bolded numbers—statistically significant results.

## Data Availability

Data used for the study can be obtained on reasonable request from the lead author.
